# Crystal structure and Hirshfeld surface analysis of the methanol solvate of sclareol, a labdane-type diterpenoid

**DOI:** 10.1107/S2056989020001474

**Published:** 2020-02-06

**Authors:** Memoona Bibi, M. Iqbal Choudhary, Sammer Yousuf

**Affiliations:** a H. E. J. Research Institute of Chemistry, International Center for Chemical and Biological Sciences, University of Karachi, Karachi-75270, Pakistan

**Keywords:** labdane diterpene, *Salvia sclarea L*, clary sage, solvate, Hirshfeld surface analysis, leishmaniasis, crystal structure

## Abstract

The title compound, C_20_H_36_O_2_·CH_3_OH [systematic name: (3*S*)-4-[(*S*)-3-hy­droxy-3-methyl­pent-4-en-1-yl]-3,4a,8,8-tetra­methyl­deca­hydro­naphthalen-3-ol methanol monosolvate], is a methanol solvate of sclareol, a diterpene oil isolated from the medicinally important medicinal herb *Salvia sclarea*, commonly known as clary sage. It crystallizes in space group *P*1 (No. 1) with *Z*′ = 2. The sclareol mol­ecule comprises two *trans*-fused cyclo­hexane rings, each having an equatorially oriented hydroxyl group, and a 3-methyl­pent-1-en-3-ol side chain. In the crystal, O_s_—H⋯O_s_, O_s_—H⋯O_m_, O_m_—H⋯O_s_ and O_m_—H⋯O_m_ (s = sclareol, m = methanol) hydrogen bonds connect neighboring mol­ecules into infinite [010] chains. The title compound exhibits weak anti-leishmanial activity (IC_50_ = 66.4 ± 1.0 µ*M* ml^−1^) against standard miltefosine (IC_50_ = 25.8 ± 0.2 µ*M* ml^−1^).

## Abstract 2

The key inter­actions in the extended structure of the title solvate are O—H⋯O hydrogen bonds, which generate [010] chains.

## Chemical context   

Sclareol, a labdane diterpene, is an important component of *Salvia sclarea L*., commonly known as clary sage, a medicinal herb mostly found in Mediterranean countries and southern Europe (Kouzi & McChesney, 1990 ▸; Acimovic *et al.*, 2018 ▸). Sclareol is also reported from *Cleome spinose* B, *Cistus creticus* C, and *Nicotiana glutinosa* S (Caniard *et al.*, 2012 ▸). Labdanes show various biological and pharmacological activities (Singh *et al.*, 1999 ▸), including anti­fungal, anti­bacterial, growth-regulating activity, and cytostatic and cytotoxic effects against HL60 human leukemic cell lines (Kouzi *et al.*, 1993 ▸; Dimas *et al.*, 2001 ▸). Sclareol is also used commercially as a fixative in perfumery and as a flavouring agent in the tobacco industry (Kouzi & McChesney, 1990 ▸).
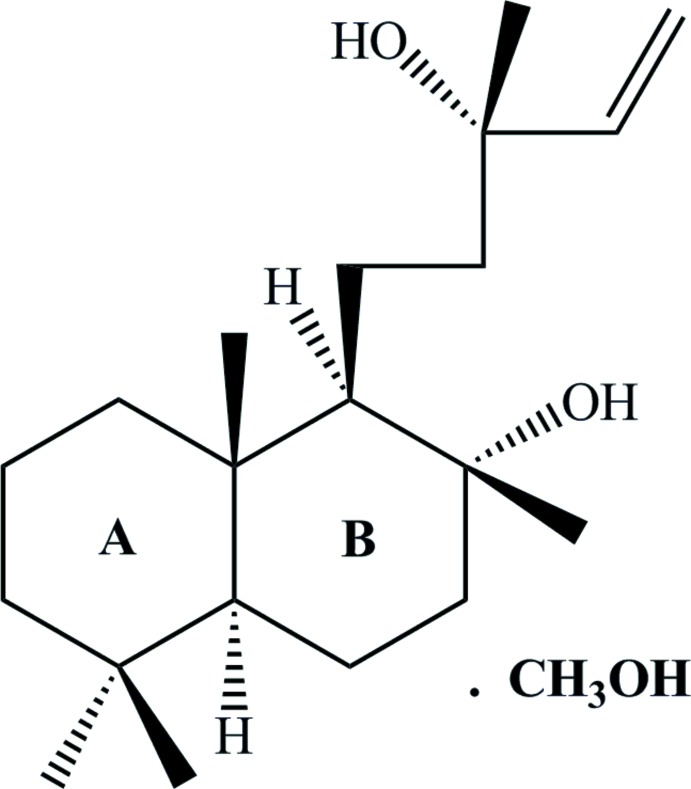


The presence of solvent mol­ecules of crystallization (Aitipamula *et al.*, 2012 ▸) can significantly influence the geometry of the respective pharmaceutical mol­ecule (Chen *et al.*, 2017 ▸). The crystal structure of sclareol (ortho­rhom­bic, space group *P*2_1_2_1_2_1_) has been described (Nagashima *et al.*, 1997 ▸). We now describe the crystal structure of the methanol solvate of sclareol (**1**), which results in a change of space group to triclinic *P*1. Leishmaniasis is a major infectious disease caused by various species of the genus *Leishmania*. Currently there is no effective drug or vaccine against leishmanicidal disease commercially available (Tavares *et al.*, 2018 ▸). In the current study, the anti-leishmanial activity of **1** was also investigated.

## Structural commentary   

**Figure 1:**
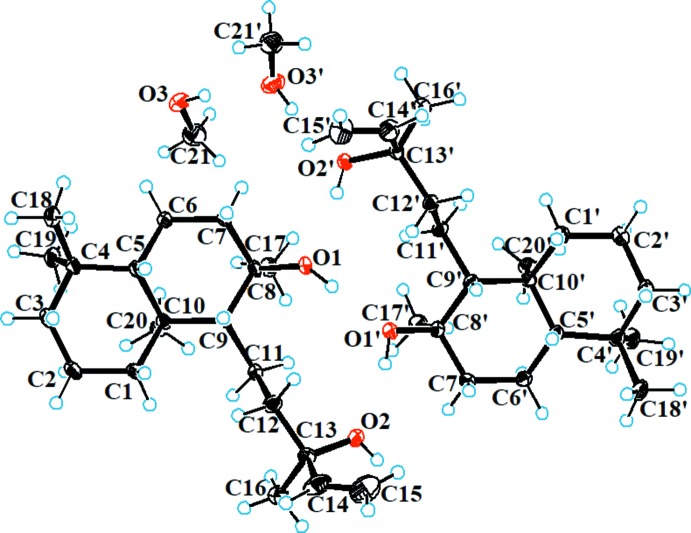
The mol­ecular structure of **1** with displacement ellipsoids drawn at the 30% probability level.

The asymmetric unit (and unit cell) of **1** consists of two independent sclareol mol­ecules and two methanol solvent mol­ecules (Fig. 1 ▸). The sclareol skeleton comprises two *trans*-fused cyclo­hexane rings, *A* (C1–C5/C10) and *B* (C5–C10), which exist in chair conformations, having puckering parameters *Q =* 0.556 (3) Å, θ *=* 3.4 (3)°, φ = 26 (4)°, and *Q* = 0.589 (3) Å, θ = 7.1 (3)°, φ = 347 (2)°, respectively. Ring *A* bears an axially oriented methyl group at C10 while ring *B* has an equatorially oriented hydroxyl group and 3-methyl­pent-1-en-3-ol side chain attached at C8 and C9, respectively. The C11—C9—C8—C17 and C11′—C9′—C8′—C17′ torsion angles of 58.7 (3) and 57.4 (3)°, respectively, indicate that the methyl group and the 3-methyl­pent-1-en-3-ol side chain are *anti* to each other in both mol­ecules. The configurations of the stereogenic centres are as follows: C5 *S*, C8 *R*, C9 *R*, C10 *S* and C13 *R*; C5′ *S*, C8′ *R*, C9′ *R*, C10′ *S* and C13′ *R*.

## Supra­molecular features and Hirshfeld surface analysis   

**Figure 2:**
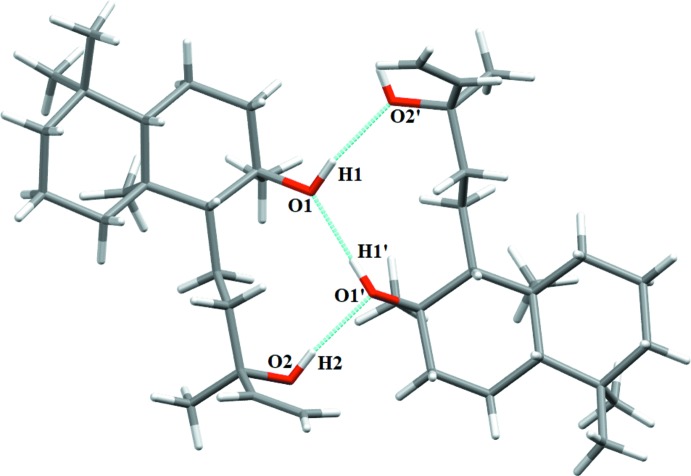
Fragment of **1** showing O—H⋯O hydrogen bonds.

**Figure 3:**
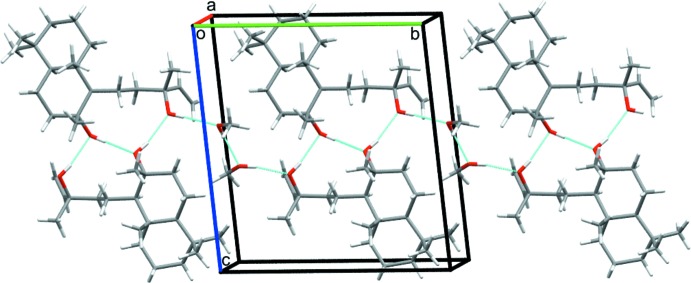
Packing diagram of **1** showing the formation of a [010] chain of mol­ecules linked by O—H⋯O hydrogen bonds (dotted lines).

The insertion of the methanol solvent into the crystal alters the previously reported ortho­rhom­bic *P*2_1_2_1_2_1_ crystal symmetry of sclareol (Nagashima *et al.*, 1997 ▸) to triclinic *P*1. The O—H⋯O hydrogen-bonding inter­actions (Table 1 ▸) including O1—H1⋯O2′, O2—H2⋯O1′ and O1′—H1′⋯O1 with H⋯*A* distances of 1.97 (4), 1.96 (5) and 1.87 (5) Å, respectively, generate 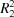
(10) ring motifs (Fig. 2 ▸). The O3 methanol solvent mol­ecule links to a sclareol host *via* an O—H⋯O hydrogen bond and the O3′ methanol mol­ecule links to the O3 methanol mol­ecule (Fig. 3 ▸). Taken together, the hydrogen bonds generate infinite [010] chains in the crystal.

**Figure 4:**
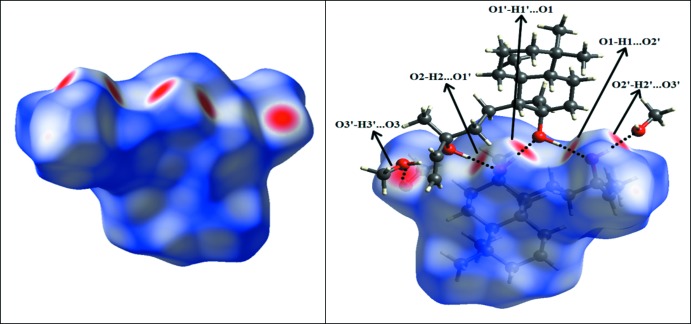
Hirshfeld surface mapped over *d*_norm_ of **1** with neighboring mol­ecules linked *via* O—H⋯O hydrogen bonds (dashed lines).

**Figure 5:**
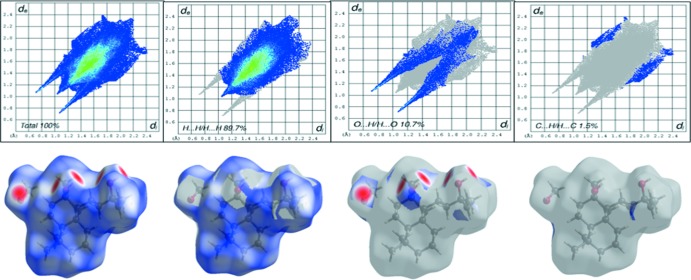
Two-dimensional Hirshfeld fingerprint plots for **1**.

The Hirshfeld surface (Spackman & Jayatilaka, 2009 ▸; Capozzi *et al.*, 2019 ▸) mapped over *d*_norm_ for **1** is shown in Fig. 4 ▸; red spots indicate the areas of the mol­ecular surfaces where strong inter­actions occur. The two-dimensional fingerprint plots (Fig. 5 ▸) indicate a dominant contribution from H⋯H contacts (89.7%) to the Hirshfeld surface; distinct spikes denote the O⋯H/H⋯O inter­actions (10.7%) while C⋯H/H⋯C contacts contribute a negligible percentage (1.5%) towards the total generated Hirshfeld surface. Views of the Hirshfeld surface mapped over shape-index and curvature are shown in the supporting information.

## *In vitro* anti-leishmanial activity   

An *in vitro* anti-leishmanial assay of compound **1** was evaluated against *L. major* promastigoates. The title compound has a relatively weak anti-leishmanial activity [(IC_50_ = 66.4 ± 1.0 µ*M* ml^−1^) against the standard miltefosine drug (IC_50_ = 25.8 ± 0.2 µ*M* ml^−1^); however, no activity was observed against tested standard penta­midine (IC_50_ = 9.24 ± 0.005 µ*M* ml^−1^) and amphotericin B (IC_50_ = 0.42 ± 0.005 µ*M* ml^−1^).

## Database survey   

A search of the Cambridge Structural Database (CSD version 5.40, update of November 2018; Groom *et al.*, 2016 ▸) gave five hits for similar diterpenoids having two *trans-*fused cyclo­hexane rings along with an equatorially oriented 3-methyl­pent-1-en-3-ol side chain, *viz*. refcodes JOBLUH (Aranda *et al.*, 1991 ▸), RULHAH (Nagashima *et al.*, 1997 ▸), KADLIK (Rodríguez *et al.*, 1989 ▸), MIFWED (Kooijman *et al.*, 2002 ▸) and MIDNIZ (Häfner *et al.*, 2018 ▸). RULHAH {systematic name: (3S)-4-(S)-3-hy­droxy-3-methyl­pent-4-en-1-yl)-3,4a,8,8-tetra­methyl­deca­hydro­naphthalen-3-ol} is the unsolvated crystal structure of sclareol. The other four compounds belong to the same class of diterpene with different substituents.

## Crystallization   

Purified sclareol was taken from the mol­ecular bank facility of the Dr Panjwani Center for Mol­ecular Medicine and Drug Research, ICCBS, University of Karachi, Pakistan. The procedure for isolation and purification has already been described (Shawl *et al.*, 1999 ▸). Crystallization was carried out in a 1:1 solvent mixture of aceto­nitrile and methanol. Colourless blocks of **1** were obtained by slow evaporation at 277 K after two weeks

## Refinement   

Crystal data, data collection and structure refinement details are summarized in Table 2 ▸. All the C-bound H atoms were located with idealized geometry and refined with C—H = 0.95–1.00 Å, having *U*_iso_(H) = 1.5*U*_eq_(CH_3_) and 1.2*U*_eq_ (CH_2_, CH). The O-bound H atoms were found in difference-Fourier maps and their positions freely refined with *U*_iso_(H) = 1.2*U*_eq_(O).

## Supplementary Material

Crystal structure: contains datablock(s) I. DOI: 10.1107/S2056989020001474/hb7890sup1.cif

Structure factors: contains datablock(s) I. DOI: 10.1107/S2056989020001474/hb7890Isup2.hkl

Hirshfeld curvature and shape index. DOI: 10.1107/S2056989020001474/hb7890sup3.docx

Supporting information file. DOI: 10.1107/S2056989020001474/hb7890Isup4.cml

CCDC reference: 1981522

Additional supporting information:  crystallographic information; 3D view; checkCIF report

## Figures and Tables

**Table 1:** Hydrogen-bond geometry (Å, °)

*D*—H⋯*A*	*D*—H	H⋯*A*	*D*⋯*A*	*D*—H⋯*A*
O1—H1⋯O2′	0.87 (4)	1.97 (4)	2.816 (2)	163 (4)
O2—H2⋯O1′	0.81 (5)	1.96 (5)	2.762 (2)	167 (4)
O3—H3⋯O2	0.88 (4)	1.89 (5)	2.744 (3)	164 (4)
O1′—H1′⋯O1	0.88 (5)	1.87 (5)	2.744 (2)	170 (4)
O2′—H2′⋯O3′^i^	0.76 (5)	2.08 (5)	2.833 (3)	169 (5)
O3′—H3′⋯O3	0.90 (5)	1.89 (5)	2.753 (3)	160 (4)

Symmetry code: (i) 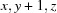.

**Table 2:** Experimental details

Crystal data	
Chemical formula	C_20_H_36_O_2_·CH_4_O
*M*_r_	340.53
Crystal system, space group	Triclinic, *P*1
Temperature (K)	100
*a*, *b*, *c* (Å)	6.1728 (3), 12.3721 (6), 13.6788 (7)
α, β, γ (°)	84.302 (2), 80.846 (2), 80.909 (2)
*V* (Å^3^)	1015.45 (9)
*Z*	2
Radiation type	Cu *K*α
μ (mm^−1^)	0.56
Crystal size (mm)	0.38 × 0.23 × 0.13

Data collection	
Diffractometer	Bruker APEXII CCD
Absorption correction	–
No. of measured, independent and observed [*I* > 2σ(*I*)] reflections	28545, 7074, 7020
*R*_int_	0.028
(sin θ/λ)_max_ (Å^−1^)	0.602

Refinement	
*R*[*F* ^2^ > 2σ(*F* ^2^)], *wR*(*F* ^2^), *S*	0.037, 0.110, 1.01
No. of reflections	7074
No. of parameters	464
No. of restraints	3
H-atom treatment	H atoms treated by a mixture of independent and constrained refinement
Δρ_max_, Δρ_min_ (e Å^−3^)	0.50, −0.21
Absolute structure parameter	0.16 (18)

Computer programs: *APEX2* and *SAINT* (Bruker, 2012 ▸), *SHELXT2014/5* (Sheldrick, 2015*a* ▸) and *SHELXL2016/6* (Sheldrick, 2015*b* ▸).

## supplementary crystallographic information

### Crystal data

**Table:**

C_20_H_36_O_2_·CH_4_O	*Z* = 2
*M**_r_* = 340.53	*F*(000) = 378
Triclinic, *P*1	*D*_x_ = 1.110 Mg m^−^^3^
*a* = 6.1728 (3) Å	Cu *K*α radiation, λ = 1.54178 Å
*b* = 12.3721 (6) Å	Cell parameters from 9050 reflections
*c* = 13.6788 (7) Å	θ = 7.8–68.3°
α = 84.302 (2)°	µ = 0.56 mm^−^^1^
β = 80.846 (2)°	*T* = 100 K
γ = 80.909 (2)°	Block, colourless
*V* = 1015.45 (9) Å^3^	0.38 × 0.23 × 0.13 mm

### Data collection

**Table:**

Bruker APEXII CCD diffractometer	*R*_int_ = 0.028
φ and ω scans	θ_max_ = 68.2°, θ_min_ = 5.1°
28545 measured reflections	*h* = −7→7
7074 independent reflections	*k* = −14→14
7020 reflections with *I* > 2σ(*I*)	*l* = −16→16

### Refinement

**Table:**

Refinement on *F*^2^	Primary atom site location: dual
Least-squares matrix: full	Hydrogen site location: mixed
*R*[*F*^2^ > 2σ(*F*^2^)] = 0.037	H atoms treated by a mixture of independent and constrained refinement
*wR*(*F*^2^) = 0.110	*w* = 1/[σ^2^(*F*_o_^2^) + (0.081*P*)^2^ + 0.2189*P*] where *P* = (*F*_o_^2^ + 2*F*_c_^2^)/3
*S* = 1.01	(Δ/σ)_max_ = 0.002
7074 reflections	Δρ_max_ = 0.50 e Å^−^^3^
464 parameters	Δρ_min_ = −0.21 e Å^−^^3^
3 restraints	

### Special details

**Table:**

Geometry. All esds (except the esd in the dihedral angle between two l.s. planes) are estimated using the full covariance matrix. The cell esds are taken into account individually in the estimation of esds in distances, angles and torsion angles; correlations between esds in cell parameters are only used when they are defined by crystal symmetry. An approximate (isotropic) treatment of cell esds is used for estimating esds involving l.s. planes.

### Fractional atomic coordinates and isotropic or equivalent isotropic displacement parameters (Å^2^)

**Table:**

	*x*	*y*	*z*	*U*_iso_*/*U*_eq_	
O1	0.4784 (3)	0.64520 (15)	0.53573 (12)	0.0254 (4)	
O2	0.5887 (3)	0.29578 (14)	0.62956 (12)	0.0168 (3)	
O3	0.5603 (4)	0.08465 (17)	0.59341 (16)	0.0356 (5)	
C1	0.3517 (4)	0.6326 (2)	0.89758 (17)	0.0193 (5)	
H1A	0.318778	0.556226	0.910076	0.023*	
H1B	0.514599	0.629395	0.881628	0.023*	
C2	0.2703 (4)	0.6926 (2)	0.99203 (17)	0.0229 (5)	
H2A	0.109176	0.691275	1.011361	0.027*	
H2B	0.346927	0.654494	1.046815	0.027*	
C3	0.3150 (4)	0.8116 (2)	0.97588 (18)	0.0242 (5)	
H3A	0.477316	0.812019	0.963476	0.029*	
H3B	0.255830	0.848734	1.037476	0.029*	
C4	0.2117 (4)	0.8776 (2)	0.88881 (17)	0.0197 (5)	
C5	0.2829 (4)	0.81065 (18)	0.79513 (16)	0.0165 (5)	
H5A	0.447652	0.807518	0.780592	0.020*	
C6	0.1973 (4)	0.86853 (19)	0.70123 (17)	0.0204 (5)	
H6A	0.036728	0.865182	0.706101	0.025*	
H6B	0.217951	0.946753	0.695765	0.025*	
C7	0.3208 (4)	0.8145 (2)	0.60876 (17)	0.0217 (5)	
H7A	0.258907	0.852136	0.549816	0.026*	
H7B	0.478688	0.824544	0.601016	0.026*	
C8	0.3068 (4)	0.69224 (19)	0.61166 (16)	0.0195 (5)	
C9	0.3730 (4)	0.63500 (18)	0.71134 (16)	0.0153 (4)	
H9A	0.529908	0.647146	0.709885	0.018*	
C10	0.2437 (4)	0.68813 (18)	0.80690 (16)	0.0152 (5)	
C11	0.3847 (4)	0.50884 (18)	0.71485 (17)	0.0165 (5)	
H11A	0.282256	0.483913	0.772988	0.020*	
H11B	0.335633	0.489148	0.654297	0.020*	
C12	0.6188 (4)	0.45017 (19)	0.72185 (16)	0.0167 (5)	
H12A	0.662724	0.467844	0.784280	0.020*	
H12B	0.720954	0.480076	0.666145	0.020*	
C13	0.6511 (4)	0.32450 (19)	0.71953 (16)	0.0168 (5)	
C14	0.8933 (4)	0.2823 (2)	0.7242 (2)	0.0250 (5)	
H14A	0.945773	0.286512	0.785170	0.030*	
C15	1.0374 (5)	0.2403 (2)	0.6516 (2)	0.0323 (6)	
H15A	0.992358	0.234497	0.589297	0.039*	
H15B	1.186820	0.215822	0.661295	0.039*	
C16	0.5052 (4)	0.2701 (2)	0.80480 (17)	0.0230 (5)	
H16A	0.348826	0.293799	0.797924	0.035*	
H16B	0.533900	0.291423	0.868129	0.035*	
H16C	0.539068	0.190152	0.803125	0.035*	
C17	0.0848 (5)	0.6728 (2)	0.58652 (19)	0.0266 (5)	
H17A	0.073983	0.698494	0.517120	0.040*	
H17B	−0.036062	0.713207	0.629984	0.040*	
H17C	0.073936	0.594183	0.596300	0.040*	
C18	−0.0400 (4)	0.9065 (2)	0.91730 (19)	0.0261 (5)	
H18A	−0.071381	0.952806	0.973438	0.039*	
H18B	−0.105549	0.838908	0.936158	0.039*	
H18C	−0.103815	0.946206	0.860520	0.039*	
C19	0.3108 (5)	0.9856 (2)	0.86890 (19)	0.0273 (6)	
H19A	0.231711	1.034786	0.821354	0.041*	
H19B	0.468106	0.970019	0.841426	0.041*	
H19C	0.295743	1.020728	0.931237	0.041*	
C20	−0.0030 (4)	0.6716 (2)	0.82343 (17)	0.0195 (5)	
H20A	−0.016327	0.601057	0.799861	0.029*	
H20B	−0.088050	0.731123	0.786519	0.029*	
H20C	−0.061023	0.672312	0.894385	0.029*	
C21	0.7310 (5)	0.0149 (2)	0.6296 (2)	0.0316 (6)	
H21A	0.787444	0.050837	0.679071	0.047*	
H21B	0.850572	−0.003342	0.574925	0.047*	
H21C	0.676596	−0.052475	0.660616	0.047*	
H1	0.527 (7)	0.698 (4)	0.496 (3)	0.047*	
H2	0.620 (7)	0.344 (4)	0.587 (3)	0.047*	
H3	0.593 (7)	0.150 (4)	0.598 (3)	0.047*	
O1'	0.6603 (3)	0.44467 (15)	0.46767 (12)	0.0212 (4)	
O2'	0.5847 (3)	0.80229 (15)	0.37901 (13)	0.0233 (4)	
O3'	0.4431 (4)	0.02360 (15)	0.42307 (15)	0.0315 (4)	
C1'	0.8272 (4)	0.4748 (2)	0.10293 (16)	0.0178 (5)	
H1'C	0.746711	0.549193	0.089278	0.021*	
H1'D	0.969576	0.483180	0.123917	0.021*	
C2'	0.8752 (4)	0.4148 (2)	0.00713 (17)	0.0225 (5)	
H2'C	0.733849	0.411160	−0.017075	0.027*	
H2'D	0.967042	0.456366	−0.044670	0.027*	
C3'	0.9966 (4)	0.2986 (2)	0.02478 (17)	0.0221 (5)	
H3'C	1.144075	0.303261	0.042423	0.027*	
H3'D	1.020401	0.261636	−0.037815	0.027*	
C4'	0.8720 (4)	0.2281 (2)	0.10727 (16)	0.0175 (5)	
C5'	0.8070 (4)	0.29321 (19)	0.20155 (15)	0.0156 (5)	
H5'B	0.951868	0.300536	0.222311	0.019*	
C6'	0.6868 (4)	0.23040 (19)	0.29028 (16)	0.0188 (5)	
H6'C	0.760689	0.153434	0.295796	0.023*	
H6'D	0.531744	0.229759	0.280080	0.023*	
C7'	0.6883 (4)	0.28425 (19)	0.38620 (16)	0.0195 (5)	
H7'C	0.605665	0.243516	0.442155	0.023*	
H7'D	0.843335	0.278028	0.398973	0.023*	
C8'	0.5866 (4)	0.40447 (19)	0.38362 (15)	0.0173 (5)	
C9'	0.6913 (4)	0.46712 (18)	0.28773 (16)	0.0153 (5)	
H9'B	0.852132	0.460276	0.294146	0.018*	
C10'	0.6878 (4)	0.41355 (19)	0.18894 (15)	0.0149 (5)	
C11'	0.6089 (4)	0.59222 (19)	0.28424 (16)	0.0171 (5)	
H11C	0.542379	0.615174	0.222839	0.021*	
H11D	0.492239	0.608286	0.341618	0.021*	
C12'	0.7963 (4)	0.65792 (19)	0.28669 (18)	0.0203 (5)	
H12C	0.908418	0.643263	0.227409	0.024*	
H12D	0.868165	0.630119	0.345828	0.024*	
C13'	0.7316 (4)	0.78318 (19)	0.28935 (17)	0.0198 (5)	
C14'	0.9418 (5)	0.8313 (2)	0.2854 (2)	0.0339 (6)	
H14B	1.040294	0.829002	0.224462	0.041*	
C15'	1.0016 (6)	0.8753 (3)	0.3560 (3)	0.0428 (7)	
H15C	0.908853	0.879598	0.418333	0.051*	
H15D	1.138537	0.903498	0.345856	0.051*	
C16'	0.6126 (5)	0.8343 (2)	0.2011 (2)	0.0326 (6)	
H16D	0.479166	0.800358	0.201777	0.049*	
H16E	0.712266	0.821499	0.138778	0.049*	
H16F	0.570633	0.913410	0.206831	0.049*	
C17'	0.3332 (4)	0.4173 (2)	0.40188 (17)	0.0229 (5)	
H17D	0.283551	0.386445	0.469007	0.034*	
H17E	0.281417	0.378218	0.353152	0.034*	
H17F	0.272408	0.495291	0.395170	0.034*	
C18'	1.0347 (4)	0.1241 (2)	0.13027 (18)	0.0242 (5)	
H18D	1.096705	0.089449	0.068545	0.036*	
H18E	0.955905	0.072601	0.176329	0.036*	
H18F	1.154937	0.144153	0.160549	0.036*	
C19'	0.6731 (4)	0.1913 (2)	0.07099 (18)	0.0236 (5)	
H19D	0.576523	0.255871	0.047117	0.035*	
H19E	0.589350	0.151320	0.125986	0.035*	
H19F	0.726912	0.143175	0.016803	0.035*	
C20'	0.4479 (4)	0.4233 (2)	0.16401 (17)	0.0190 (5)	
H20D	0.360754	0.491371	0.187734	0.028*	
H20E	0.378987	0.360391	0.196612	0.028*	
H20F	0.452926	0.424146	0.091969	0.028*	
C21'	0.2414 (5)	0.0939 (3)	0.4111 (2)	0.0349 (6)	
H21D	0.118965	0.066723	0.456538	0.052*	
H21E	0.213118	0.094828	0.342464	0.052*	
H21F	0.253422	0.168387	0.425907	0.052*	
H1'	0.586 (7)	0.507 (4)	0.488 (3)	0.052*	
H2'	0.564 (7)	0.862 (4)	0.391 (3)	0.052*	
H3'	0.514 (7)	0.038 (4)	0.472 (3)	0.052*	

### Atomic displacement parameters (Å^2^)

**Table:**

	*U*^11^	*U*^22^	*U*^33^	*U*^12^	*U*^13^	*U*^23^
O1	0.0414 (10)	0.0174 (9)	0.0142 (8)	−0.0050 (8)	0.0070 (7)	−0.0023 (7)
O2	0.0235 (8)	0.0160 (8)	0.0129 (7)	−0.0058 (6)	−0.0050 (6)	−0.0015 (6)
O3	0.0511 (12)	0.0199 (10)	0.0393 (11)	−0.0047 (9)	−0.0177 (9)	−0.0021 (8)
C1	0.0202 (11)	0.0184 (12)	0.0194 (11)	−0.0038 (9)	−0.0013 (9)	−0.0024 (9)
C2	0.0288 (12)	0.0249 (14)	0.0149 (11)	−0.0047 (10)	−0.0032 (9)	−0.0002 (9)
C3	0.0304 (13)	0.0267 (14)	0.0166 (11)	−0.0072 (10)	0.0002 (9)	−0.0074 (9)
C4	0.0237 (12)	0.0162 (12)	0.0183 (11)	−0.0054 (9)	0.0045 (9)	−0.0047 (9)
C5	0.0170 (10)	0.0150 (12)	0.0170 (11)	−0.0036 (9)	0.0021 (8)	−0.0041 (9)
C6	0.0277 (12)	0.0134 (11)	0.0185 (11)	−0.0026 (9)	0.0016 (9)	−0.0011 (9)
C7	0.0321 (13)	0.0165 (12)	0.0150 (10)	−0.0047 (10)	0.0014 (9)	0.0000 (8)
C8	0.0280 (12)	0.0151 (12)	0.0146 (10)	−0.0040 (9)	0.0007 (9)	−0.0026 (8)
C9	0.0163 (10)	0.0135 (11)	0.0162 (11)	−0.0042 (8)	−0.0002 (8)	−0.0021 (8)
C10	0.0160 (10)	0.0150 (12)	0.0141 (10)	−0.0040 (9)	0.0009 (8)	−0.0009 (8)
C11	0.0197 (11)	0.0129 (12)	0.0175 (10)	−0.0055 (9)	−0.0008 (8)	−0.0016 (8)
C12	0.0181 (11)	0.0169 (12)	0.0165 (10)	−0.0055 (9)	−0.0032 (8)	−0.0032 (8)
C13	0.0208 (11)	0.0186 (12)	0.0127 (10)	−0.0033 (9)	−0.0058 (8)	−0.0035 (8)
C14	0.0249 (12)	0.0241 (13)	0.0284 (12)	−0.0012 (10)	−0.0124 (10)	−0.0036 (10)
C15	0.0232 (12)	0.0369 (16)	0.0384 (15)	−0.0015 (11)	−0.0074 (11)	−0.0102 (12)
C16	0.0341 (13)	0.0200 (12)	0.0158 (11)	−0.0074 (10)	−0.0037 (9)	0.0004 (9)
C17	0.0359 (14)	0.0229 (14)	0.0224 (12)	−0.0025 (11)	−0.0118 (10)	0.0001 (10)
C18	0.0260 (12)	0.0245 (13)	0.0254 (12)	−0.0019 (10)	0.0056 (10)	−0.0075 (10)
C19	0.0369 (14)	0.0220 (13)	0.0238 (12)	−0.0124 (11)	0.0061 (10)	−0.0094 (10)
C20	0.0186 (11)	0.0190 (12)	0.0209 (11)	−0.0044 (9)	−0.0004 (8)	−0.0021 (9)
C21	0.0343 (14)	0.0283 (15)	0.0334 (14)	−0.0091 (12)	−0.0052 (11)	−0.0009 (11)
O1'	0.0340 (9)	0.0178 (9)	0.0120 (7)	−0.0034 (7)	−0.0026 (6)	−0.0038 (6)
O2'	0.0325 (10)	0.0171 (9)	0.0199 (8)	−0.0084 (7)	0.0044 (7)	−0.0043 (7)
O3'	0.0474 (12)	0.0188 (10)	0.0290 (10)	−0.0037 (8)	−0.0078 (9)	−0.0034 (7)
C1'	0.0219 (11)	0.0194 (12)	0.0127 (10)	−0.0077 (9)	−0.0011 (8)	0.0017 (8)
C2'	0.0290 (12)	0.0247 (13)	0.0133 (10)	−0.0086 (10)	0.0013 (9)	0.0012 (9)
C3'	0.0279 (12)	0.0261 (14)	0.0119 (10)	−0.0066 (10)	0.0025 (9)	−0.0029 (9)
C4'	0.0222 (11)	0.0189 (12)	0.0113 (10)	−0.0049 (9)	0.0003 (8)	−0.0025 (8)
C5'	0.0198 (11)	0.0185 (12)	0.0097 (10)	−0.0058 (9)	−0.0019 (8)	−0.0020 (8)
C6'	0.0277 (12)	0.0146 (12)	0.0142 (10)	−0.0062 (9)	0.0001 (9)	−0.0023 (8)
C7'	0.0316 (13)	0.0161 (12)	0.0109 (10)	−0.0060 (10)	−0.0007 (9)	−0.0008 (8)
C8'	0.0235 (11)	0.0190 (12)	0.0100 (10)	−0.0072 (9)	0.0008 (8)	−0.0031 (8)
C9'	0.0180 (10)	0.0158 (12)	0.0121 (10)	−0.0042 (9)	−0.0010 (8)	−0.0009 (8)
C10'	0.0167 (10)	0.0177 (12)	0.0117 (10)	−0.0070 (9)	−0.0012 (8)	−0.0016 (8)
C11'	0.0219 (11)	0.0162 (12)	0.0130 (10)	−0.0039 (9)	−0.0001 (8)	−0.0022 (8)
C12'	0.0200 (11)	0.0162 (12)	0.0242 (12)	−0.0058 (9)	0.0005 (9)	−0.0010 (9)
C13'	0.0236 (11)	0.0172 (12)	0.0178 (11)	−0.0076 (9)	0.0029 (9)	0.0002 (8)
C14'	0.0295 (14)	0.0215 (14)	0.0508 (18)	−0.0123 (11)	0.0013 (12)	−0.0012 (12)
C15'	0.0358 (15)	0.0369 (18)	0.060 (2)	−0.0106 (13)	−0.0124 (14)	−0.0064 (14)
C16'	0.0524 (17)	0.0225 (14)	0.0240 (13)	−0.0079 (12)	−0.0074 (12)	0.0005 (10)
C17'	0.0238 (12)	0.0243 (13)	0.0194 (11)	−0.0081 (10)	0.0059 (9)	−0.0030 (9)
C18'	0.0302 (13)	0.0223 (13)	0.0184 (11)	0.0011 (10)	−0.0011 (9)	−0.0053 (9)
C19'	0.0327 (13)	0.0238 (13)	0.0173 (11)	−0.0101 (11)	−0.0038 (10)	−0.0057 (9)
C20'	0.0189 (11)	0.0206 (12)	0.0187 (10)	−0.0053 (9)	−0.0035 (8)	−0.0026 (8)
C21'	0.0336 (14)	0.0345 (17)	0.0362 (15)	−0.0092 (12)	−0.0007 (12)	−0.0013 (12)

### Geometric parameters (Å, º)

**Table:**

O1—C8	1.454 (3)	O1'—C8'	1.458 (3)
O1—H1	0.87 (4)	O1'—H1'	0.88 (5)
O2—C13	1.439 (3)	O2'—C13'	1.421 (3)
O2—H2	0.81 (5)	O2'—H2'	0.76 (5)
O3—C21	1.376 (4)	O3'—C21'	1.424 (4)
O3—H3	0.88 (4)	O3'—H3'	0.90 (5)
C1—C2	1.530 (3)	C1'—C2'	1.535 (3)
C1—C10	1.551 (3)	C1'—C10'	1.551 (3)
C1—H1A	0.9900	C1'—H1'C	0.9900
C1—H1B	0.9900	C1'—H1'D	0.9900
C2—C3	1.528 (4)	C2'—C3'	1.528 (4)
C2—H2A	0.9900	C2'—H2'C	0.9900
C2—H2B	0.9900	C2'—H2'D	0.9900
C3—C4	1.542 (4)	C3'—C4'	1.538 (3)
C3—H3A	0.9900	C3'—H3'C	0.9900
C3—H3B	0.9900	C3'—H3'D	0.9900
C4—C18	1.534 (3)	C4'—C18'	1.541 (4)
C4—C19	1.538 (3)	C4'—C19'	1.542 (3)
C4—C5	1.562 (3)	C4'—C5'	1.553 (3)
C5—C6	1.535 (3)	C5'—C6'	1.528 (3)
C5—C10	1.561 (3)	C5'—C10'	1.559 (3)
C5—H5A	1.0000	C5'—H5'B	1.0000
C6—C7	1.526 (3)	C6'—C7'	1.531 (3)
C6—H6A	0.9900	C6'—H6'C	0.9900
C6—H6B	0.9900	C6'—H6'D	0.9900
C7—C8	1.524 (3)	C7'—C8'	1.519 (3)
C7—H7A	0.9900	C7'—H7'C	0.9900
C7—H7B	0.9900	C7'—H7'D	0.9900
C8—C17	1.524 (4)	C8'—C17'	1.529 (3)
C8—C9	1.556 (3)	C8'—C9'	1.557 (3)
C9—C11	1.547 (3)	C9'—C11'	1.550 (3)
C9—C10	1.565 (3)	C9'—C10'	1.568 (3)
C9—H9A	1.0000	C9'—H9'B	1.0000
C10—C20	1.546 (3)	C10'—C20'	1.555 (3)
C11—C12	1.524 (3)	C11'—C12'	1.522 (3)
C11—H11A	0.9900	C11'—H11C	0.9900
C11—H11B	0.9900	C11'—H11D	0.9900
C12—C13	1.539 (3)	C12'—C13'	1.541 (3)
C12—H12A	0.9900	C12'—H12C	0.9900
C12—H12B	0.9900	C12'—H12D	0.9900
C13—C14	1.513 (3)	C13'—C14'	1.502 (3)
C13—C16	1.525 (3)	C13'—C16'	1.543 (4)
C14—C15	1.313 (4)	C14'—C15'	1.283 (5)
C14—H14A	0.9500	C14'—H14B	0.9500
C15—H15A	0.9500	C15'—H15C	0.9500
C15—H15B	0.9500	C15'—H15D	0.9500
C16—H16A	0.9800	C16'—H16D	0.9800
C16—H16B	0.9800	C16'—H16E	0.9800
C16—H16C	0.9800	C16'—H16F	0.9800
C17—H17A	0.9800	C17'—H17D	0.9800
C17—H17B	0.9800	C17'—H17E	0.9800
C17—H17C	0.9800	C17'—H17F	0.9800
C18—H18A	0.9800	C18'—H18D	0.9800
C18—H18B	0.9800	C18'—H18E	0.9800
C18—H18C	0.9800	C18'—H18F	0.9800
C19—H19A	0.9800	C19'—H19D	0.9800
C19—H19B	0.9800	C19'—H19E	0.9800
C19—H19C	0.9800	C19'—H19F	0.9800
C20—H20A	0.9800	C20'—H20D	0.9800
C20—H20B	0.9800	C20'—H20E	0.9800
C20—H20C	0.9800	C20'—H20F	0.9800
C21—H21A	0.9800	C21'—H21D	0.9800
C21—H21B	0.9800	C21'—H21E	0.9800
C21—H21C	0.9800	C21'—H21F	0.9800
			
C8—O1—H1	109 (3)	C8'—O1'—H1'	116 (3)
C13—O2—H2	106 (3)	C13'—O2'—H2'	113 (3)
C21—O3—H3	104 (3)	C21'—O3'—H3'	116 (3)
C2—C1—C10	113.11 (19)	C2'—C1'—C10'	112.77 (19)
C2—C1—H1A	109.0	C2'—C1'—H1'C	109.0
C10—C1—H1A	109.0	C10'—C1'—H1'C	109.0
C2—C1—H1B	109.0	C2'—C1'—H1'D	109.0
C10—C1—H1B	109.0	C10'—C1'—H1'D	109.0
H1A—C1—H1B	107.8	H1'C—C1'—H1'D	107.8
C3—C2—C1	110.86 (19)	C3'—C2'—C1'	111.04 (19)
C3—C2—H2A	109.5	C3'—C2'—H2'C	109.4
C1—C2—H2A	109.5	C1'—C2'—H2'C	109.4
C3—C2—H2B	109.5	C3'—C2'—H2'D	109.4
C1—C2—H2B	109.5	C1'—C2'—H2'D	109.4
H2A—C2—H2B	108.1	H2'C—C2'—H2'D	108.0
C2—C3—C4	113.9 (2)	C2'—C3'—C4'	113.7 (2)
C2—C3—H3A	108.8	C2'—C3'—H3'C	108.8
C4—C3—H3A	108.8	C4'—C3'—H3'C	108.8
C2—C3—H3B	108.8	C2'—C3'—H3'D	108.8
C4—C3—H3B	108.8	C4'—C3'—H3'D	108.8
H3A—C3—H3B	107.7	H3'C—C3'—H3'D	107.7
C18—C4—C19	107.9 (2)	C3'—C4'—C18'	107.01 (19)
C18—C4—C3	110.5 (2)	C3'—C4'—C19'	110.70 (18)
C19—C4—C3	107.2 (2)	C18'—C4'—C19'	107.6 (2)
C18—C4—C5	114.3 (2)	C3'—C4'—C5'	108.73 (18)
C19—C4—C5	108.63 (18)	C18'—C4'—C5'	108.96 (18)
C3—C4—C5	108.15 (19)	C19'—C4'—C5'	113.63 (19)
C6—C5—C10	110.12 (18)	C6'—C5'—C4'	113.84 (18)
C6—C5—C4	113.92 (19)	C6'—C5'—C10'	110.47 (18)
C10—C5—C4	117.09 (17)	C4'—C5'—C10'	117.57 (17)
C6—C5—H5A	104.8	C6'—C5'—H5'B	104.5
C10—C5—H5A	104.8	C4'—C5'—H5'B	104.5
C4—C5—H5A	104.8	C10'—C5'—H5'B	104.5
C7—C6—C5	110.79 (19)	C5'—C6'—C7'	110.36 (18)
C7—C6—H6A	109.5	C5'—C6'—H6'C	109.6
C5—C6—H6A	109.5	C7'—C6'—H6'C	109.6
C7—C6—H6B	109.5	C5'—C6'—H6'D	109.6
C5—C6—H6B	109.5	C7'—C6'—H6'D	109.6
H6A—C6—H6B	108.1	H6'C—C6'—H6'D	108.1
C8—C7—C6	113.62 (18)	C8'—C7'—C6'	113.70 (19)
C8—C7—H7A	108.8	C8'—C7'—H7'C	108.8
C6—C7—H7A	108.8	C6'—C7'—H7'C	108.8
C8—C7—H7B	108.8	C8'—C7'—H7'D	108.8
C6—C7—H7B	108.8	C6'—C7'—H7'D	108.8
H7A—C7—H7B	107.7	H7'C—C7'—H7'D	107.7
O1—C8—C17	107.19 (19)	O1'—C8'—C7'	103.63 (18)
O1—C8—C7	107.78 (18)	O1'—C8'—C17'	108.14 (18)
C17—C8—C7	111.3 (2)	C7'—C8'—C17'	111.31 (19)
O1—C8—C9	104.60 (18)	O1'—C8'—C9'	107.29 (17)
C17—C8—C9	116.24 (19)	C7'—C8'—C9'	109.75 (18)
C7—C8—C9	109.14 (19)	C17'—C8'—C9'	115.90 (19)
C11—C9—C8	111.82 (18)	C11'—C9'—C8'	112.55 (18)
C11—C9—C10	114.56 (18)	C11'—C9'—C10'	114.24 (17)
C8—C9—C10	115.04 (18)	C8'—C9'—C10'	114.75 (17)
C11—C9—H9A	104.7	C11'—C9'—H9'B	104.6
C8—C9—H9A	104.7	C8'—C9'—H9'B	104.6
C10—C9—H9A	104.7	C10'—C9'—H9'B	104.6
C20—C10—C1	108.67 (17)	C1'—C10'—C20'	108.17 (18)
C20—C10—C5	114.51 (18)	C1'—C10'—C5'	107.80 (18)
C1—C10—C5	107.27 (17)	C20'—C10'—C5'	114.18 (18)
C20—C10—C9	111.51 (18)	C1'—C10'—C9'	108.48 (17)
C1—C10—C9	108.40 (18)	C20'—C10'—C9'	111.51 (18)
C5—C10—C9	106.25 (16)	C5'—C10'—C9'	106.53 (16)
C12—C11—C9	111.66 (18)	C12'—C11'—C9'	111.79 (19)
C12—C11—H11A	109.3	C12'—C11'—H11C	109.3
C9—C11—H11A	109.3	C9'—C11'—H11C	109.3
C12—C11—H11B	109.3	C12'—C11'—H11D	109.3
C9—C11—H11B	109.3	C9'—C11'—H11D	109.3
H11A—C11—H11B	107.9	H11C—C11'—H11D	107.9
C11—C12—C13	115.76 (18)	C11'—C12'—C13'	116.4 (2)
C11—C12—H12A	108.3	C11'—C12'—H12C	108.2
C13—C12—H12A	108.3	C13'—C12'—H12C	108.2
C11—C12—H12B	108.3	C11'—C12'—H12D	108.2
C13—C12—H12B	108.3	C13'—C12'—H12D	108.2
H12A—C12—H12B	107.4	H12C—C12'—H12D	107.3
O2—C13—C14	110.35 (19)	O2'—C13'—C14'	112.2 (2)
O2—C13—C16	106.17 (17)	O2'—C13'—C12'	107.08 (18)
C14—C13—C16	110.3 (2)	C14'—C13'—C12'	107.3 (2)
O2—C13—C12	109.85 (17)	O2'—C13'—C16'	108.7 (2)
C14—C13—C12	107.79 (19)	C14'—C13'—C16'	109.4 (2)
C16—C13—C12	112.37 (19)	C12'—C13'—C16'	112.2 (2)
C15—C14—C13	125.8 (2)	C15'—C14'—C13'	126.5 (3)
C15—C14—H14A	117.1	C15'—C14'—H14B	116.7
C13—C14—H14A	117.1	C13'—C14'—H14B	116.7
C14—C15—H15A	120.0	C14'—C15'—H15C	120.0
C14—C15—H15B	120.0	C14'—C15'—H15D	120.0
H15A—C15—H15B	120.0	H15C—C15'—H15D	120.0
C13—C16—H16A	109.5	C13'—C16'—H16D	109.5
C13—C16—H16B	109.5	C13'—C16'—H16E	109.5
H16A—C16—H16B	109.5	H16D—C16'—H16E	109.5
C13—C16—H16C	109.5	C13'—C16'—H16F	109.5
H16A—C16—H16C	109.5	H16D—C16'—H16F	109.5
H16B—C16—H16C	109.5	H16E—C16'—H16F	109.5
C8—C17—H17A	109.5	C8'—C17'—H17D	109.5
C8—C17—H17B	109.5	C8'—C17'—H17E	109.5
H17A—C17—H17B	109.5	H17D—C17'—H17E	109.5
C8—C17—H17C	109.5	C8'—C17'—H17F	109.5
H17A—C17—H17C	109.5	H17D—C17'—H17F	109.5
H17B—C17—H17C	109.5	H17E—C17'—H17F	109.5
C4—C18—H18A	109.5	C4'—C18'—H18D	109.5
C4—C18—H18B	109.5	C4'—C18'—H18E	109.5
H18A—C18—H18B	109.5	H18D—C18'—H18E	109.5
C4—C18—H18C	109.5	C4'—C18'—H18F	109.5
H18A—C18—H18C	109.5	H18D—C18'—H18F	109.5
H18B—C18—H18C	109.5	H18E—C18'—H18F	109.5
C4—C19—H19A	109.5	C4'—C19'—H19D	109.5
C4—C19—H19B	109.5	C4'—C19'—H19E	109.5
H19A—C19—H19B	109.5	H19D—C19'—H19E	109.5
C4—C19—H19C	109.5	C4'—C19'—H19F	109.5
H19A—C19—H19C	109.5	H19D—C19'—H19F	109.5
H19B—C19—H19C	109.5	H19E—C19'—H19F	109.5
C10—C20—H20A	109.5	C10'—C20'—H20D	109.5
C10—C20—H20B	109.5	C10'—C20'—H20E	109.5
H20A—C20—H20B	109.5	H20D—C20'—H20E	109.5
C10—C20—H20C	109.5	C10'—C20'—H20F	109.5
H20A—C20—H20C	109.5	H20D—C20'—H20F	109.5
H20B—C20—H20C	109.5	H20E—C20'—H20F	109.5
O3—C21—H21A	109.5	O3'—C21'—H21D	109.5
O3—C21—H21B	109.5	O3'—C21'—H21E	109.5
H21A—C21—H21B	109.5	H21D—C21'—H21E	109.5
O3—C21—H21C	109.5	O3'—C21'—H21F	109.5
H21A—C21—H21C	109.5	H21D—C21'—H21F	109.5
H21B—C21—H21C	109.5	H21E—C21'—H21F	109.5
			
C10—C1—C2—C3	−58.0 (3)	C10'—C1'—C2'—C3'	−57.9 (3)
C1—C2—C3—C4	56.6 (3)	C1'—C2'—C3'—C4'	56.7 (3)
C2—C3—C4—C18	74.2 (3)	C2'—C3'—C4'—C18'	−168.60 (19)
C2—C3—C4—C19	−168.4 (2)	C2'—C3'—C4'—C19'	74.4 (3)
C2—C3—C4—C5	−51.5 (3)	C2'—C3'—C4'—C5'	−51.0 (3)
C18—C4—C5—C6	58.4 (3)	C3'—C4'—C5'—C6'	−178.50 (19)
C19—C4—C5—C6	−62.2 (3)	C18'—C4'—C5'—C6'	−62.2 (2)
C3—C4—C5—C6	−178.2 (2)	C19'—C4'—C5'—C6'	57.7 (3)
C18—C4—C5—C10	−72.1 (3)	C3'—C4'—C5'—C10'	50.1 (2)
C19—C4—C5—C10	167.3 (2)	C18'—C4'—C5'—C10'	166.36 (19)
C3—C4—C5—C10	51.3 (3)	C19'—C4'—C5'—C10'	−73.7 (3)
C10—C5—C6—C7	−61.2 (2)	C4'—C5'—C6'—C7'	163.7 (2)
C4—C5—C6—C7	164.94 (19)	C10'—C5'—C6'—C7'	−61.4 (2)
C5—C6—C7—C8	56.9 (3)	C5'—C6'—C7'—C8'	56.8 (3)
C6—C7—C8—O1	−163.9 (2)	C6'—C7'—C8'—O1'	−164.87 (18)
C6—C7—C8—C17	78.8 (3)	C6'—C7'—C8'—C17'	79.1 (2)
C6—C7—C8—C9	−50.8 (3)	C6'—C7'—C8'—C9'	−50.5 (3)
O1—C8—C9—C11	−59.3 (2)	O1'—C8'—C9'—C11'	−63.5 (2)
C17—C8—C9—C11	58.7 (3)	C7'—C8'—C9'—C11'	−175.43 (19)
C7—C8—C9—C11	−174.42 (19)	C17'—C8'—C9'—C11'	57.4 (3)
O1—C8—C9—C10	167.77 (17)	O1'—C8'—C9'—C10'	163.62 (18)
C17—C8—C9—C10	−74.3 (3)	C7'—C8'—C9'—C10'	51.7 (2)
C7—C8—C9—C10	52.6 (2)	C17'—C8'—C9'—C10'	−75.5 (3)
C2—C1—C10—C20	−70.2 (2)	C2'—C1'—C10'—C20'	−70.8 (2)
C2—C1—C10—C5	54.1 (2)	C2'—C1'—C10'—C5'	53.2 (2)
C2—C1—C10—C9	168.48 (18)	C2'—C1'—C10'—C9'	168.13 (19)
C6—C5—C10—C20	−64.1 (2)	C6'—C5'—C10'—C1'	175.93 (17)
C4—C5—C10—C20	68.2 (3)	C4'—C5'—C10'—C1'	−51.1 (2)
C6—C5—C10—C1	175.24 (18)	C6'—C5'—C10'—C20'	−63.9 (2)
C4—C5—C10—C1	−52.5 (2)	C4'—C5'—C10'—C20'	69.1 (2)
C6—C5—C10—C9	59.5 (2)	C6'—C5'—C10'—C9'	59.7 (2)
C4—C5—C10—C9	−168.29 (18)	C4'—C5'—C10'—C9'	−167.37 (17)
C11—C9—C10—C20	−63.2 (2)	C11'—C9'—C10'—C1'	56.2 (2)
C8—C9—C10—C20	68.4 (2)	C8'—C9'—C10'—C1'	−171.73 (19)
C11—C9—C10—C1	56.4 (2)	C11'—C9'—C10'—C20'	−62.9 (2)
C8—C9—C10—C1	−171.97 (18)	C8'—C9'—C10'—C20'	69.2 (2)
C11—C9—C10—C5	171.39 (18)	C11'—C9'—C10'—C5'	171.96 (17)
C8—C9—C10—C5	−57.0 (2)	C8'—C9'—C10'—C5'	−55.9 (2)
C8—C9—C11—C12	116.6 (2)	C8'—C9'—C11'—C12'	115.7 (2)
C10—C9—C11—C12	−110.2 (2)	C10'—C9'—C11'—C12'	−111.2 (2)
C9—C11—C12—C13	−176.52 (17)	C9'—C11'—C12'—C13'	−176.76 (18)
C11—C12—C13—O2	58.1 (2)	C11'—C12'—C13'—O2'	62.6 (3)
C11—C12—C13—C14	178.38 (19)	C11'—C12'—C13'—C14'	−176.7 (2)
C11—C12—C13—C16	−59.8 (2)	C11'—C12'—C13'—C16'	−56.6 (3)
O2—C13—C14—C15	9.7 (4)	O2'—C13'—C14'—C15'	7.0 (4)
C16—C13—C14—C15	126.8 (3)	C12'—C13'—C14'—C15'	−110.4 (3)
C12—C13—C14—C15	−110.2 (3)	C16'—C13'—C14'—C15'	127.7 (3)

### Hydrogen-bond geometry (Å, º)

**Table:**

*D*—H···*A*	*D*—H	H···*A*	*D*···*A*	*D*—H···*A*
O1—H1···O2′	0.87 (4)	1.97 (4)	2.816 (2)	163 (4)
O2—H2···O1′	0.81 (5)	1.96 (5)	2.762 (2)	167 (4)
O3—H3···O2	0.88 (4)	1.89 (5)	2.744 (3)	164 (4)
O1′—H1′···O1	0.88 (5)	1.87 (5)	2.744 (2)	170 (4)
O2′—H2′···O3′^i^	0.76 (5)	2.08 (5)	2.833 (3)	169 (5)
O3′—H3′···O3	0.90 (5)	1.89 (5)	2.753 (3)	160 (4)

Symmetry code: (i) *x*, *y*+1, *z*.
