# Supplementary figures and images for: Crystal structure and Hirshfeld surface analysis of the methanol solvate of sclareol, a labdane-type diterpenoid
Source: Acta Crystallogr E Crystallogr Commun. 2020 Feb 6;76(Pt 3):294–7. doi: 10.1107/S2056989020001474 (PMC7057370; doi:10.1107/S2056989020001474)

| **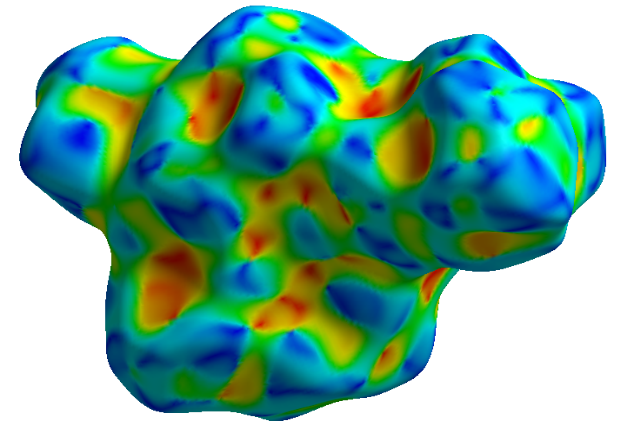**  **a** | **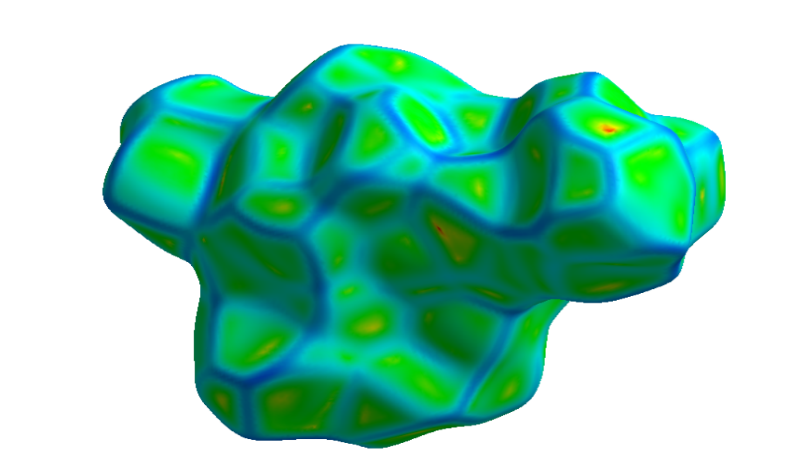**  **b** |
| --- | --- |

**Figure 6:** shape-index (a) Curvedness (b) mapped over Hirshfeld surface for title compound.

Supplement: Supplementary file 3 [file e-76-00294-sup3.docx]
